# Seeing and sensing single G protein-coupled receptors by atomic force microscopy

**DOI:** 10.1016/j.ceb.2018.10.006

**Published:** 2019-04

**Authors:** K Tanuj Sapra, Patrizia M Spoerri, Andreas Engel, David Alsteens, Daniel J Müller

**Affiliations:** 1 ETH Zürich, Department of Biosystems Science and Engineering, Mattenstrasse 26, 4058 Basel, Switzerland; 2 Louvain Institute of Biomolecular Science and Technology, Université catholique de Louvain, Croix du Sud, 4-5, bte L7.07.07., B-1348 Louvain-la-Neuve, Belgium

## Abstract

G protein-coupled receptors (GPCRs) relay extracellular information across cell membranes through a continuum of conformations that are not always captured in structures. Hence, complementary approaches are required to quantify the physical and chemical properties of the dynamic conformations linking to GPCR function. Atomic force microscopy (AFM)-based high-resolution imaging and force spectroscopy are unique methods to scrutinize GPCRs and to sense their interactions. Here, we exemplify recent AFM-based applications to directly observe the supramolecular assembly of GPCRs in native membranes, to measure the ligand-binding free-energy landscape, and how interactions modulate the structural properties of GPCRs. Common trends in GPCR function are beginning to emerge. We envision that technical developments in combining AFM with superresolution fluorescence imaging will provide insights into how cellular states modulate GPCRs and *vice versa*.

## Highlights

• G protein-coupled receptors (GPCRs) regulate key physiological processes and are therefore important drug targets.
• Complementary approaches are required to obtain mechanistic insight into the structure-function relationship of GPCRs.
• AFM-based approaches allow various properties of GPCRs to be directly observed and quantified in physiological conditions.
• FD-based AFM can image single GPCRs at high-resolution and quantify the free-energy landscape of ligand-binding.
• AFM-based force spectroscopy interrogates the physical and chemical properties of GPCRs.

**Current Opinion in Cell Biology** 2019, **57**:25–32

This review comes from a themed issue on **Cell signalling**

Edited by **Wouter H Moolenaar** and **Tamás Balla**

For a complete overview see the Issue and the Editorial

Available online 6th November 2018

**https://doi.org/10.1016/j.ceb.2018.10.006**

0955-0674/© 2018 The Authors. Published by Elsevier Ltd. This is an open access article under the CC BY license (http://creativecommons.org/licenses/by/4.0/).

## Introduction

G protein-coupled receptors (GPCRs) represent a versatile family of more than 800 transmembrane proteins (TMPs) [1] that sense and respond to a wide range of extracellular physical and chemical stimuli including light, neurotransmitters, odorants, hormones, and chemokines. GPCRs transmit extracellular information into the cell through a complex orchestration of conformational changes that activate and terminate intracellular signaling cascades with the help of G proteins and arrestins, respectively [2]. Thereby GPCRs have evolved to regulate sensory responses to the environment (vision, taste, and smell), to control blood pressure and heart rate, to modulate immune system activity, and to warrant homeostasis. Because of their importance in defining the basic human physiology and their implication in human diseases including Alzheimer’s, Parkinson’s and Huntington’s diseases [3], the majority of drug molecules are designed to target GPCRs [4].

The first X-ray structure of rhodopsin provided deep insights into the seven-transmembrane-domain architecture of GPCRs [5^••^]. Recent years have seen a surge in the number of GPCR structures bound to ligands, transducers, stabilized by nanobodies and mutations [6,7^••^,^8^, ^9^, ^10^, ^11^,12^••^,^13^] highlighting the common features in GPCR structure, signaling and function [2,14]. Such high-resolution structures are snapshots of discrete states of dynamic protein complexes [15]. Ligand-binding, GPCR activation and inactivation are not binary ‘on-off’ processes but highly dynamic with conformational transitioning between energetic states on a rough energy landscape [16,17]. To better understand the molecular details of how GPCR conformations are modulated by ligand-binding or GPCR-transducer complex formation, complementary techniques need to be developed and applied.

**Figure 1:**
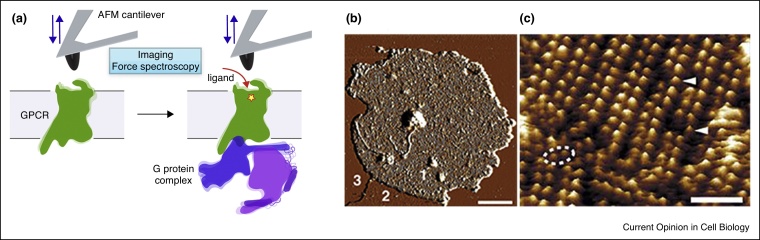
G protein-coupled receptors in native membranes. **(a)** Schematic summarizing the current and future applications of AFM imaging and force spectroscopy applied to characterize the structure and function of GPCRs in physiologically relevant conditions (buffer solution, temperature, and native or synthethic membranes). Combining the AFM imaging and force spectroscopy modes allow to directly visualize the conformation and assembly of single GPCRs and to simultaneously sense and localize individual ligand-binding events. Alternatively, the force spectroscopy mode may be used to quantify and localize intramolecular and intermolecular interactions that stabilize various functional states of the GPCR. Such functional states may be introduced by ligand-binding, mutations, lipid compositions or the presence of the G protein complex. As discussed in this review, AFM imaging and force spectroscopy of various GPCRs have been performed in the inactive and active states (with and without ligands). However, monitoring GPCR activation and interactions in presence of G protein complex remains an area for future investigations. **(b)** Example of imaging GPCRs in their physiologically relevant environment by AFM. Native rod outer segment (ROS) disc membrane adsorbed onto mica and imaged by contact mode AFM. Discs burst open upon exposure to osmotic shock to expose the cytoplasmic side (1). An area of empty lipid membrane (2) and the mica support (3) are also visible. Scale bar, 200 nm. **(c)** High-resolution AFM imaging of the membrane showing single rhodopsin molecules (white arrow heads) assembled as dimers (dashed ellipse) and arranged in rows [18^••^]. Scale bar, 15 nm. Both images were recorded in buffer solution [18^••^].

Here we highlight atomic force microscopy (AFM)-based high-resolution (<1–2 nm) imaging and single-molecule force spectroscopy (SMFS) methods as unique complementary approaches to delve into the details of the complex GPCR structure and function relationships (Figure 1a). We overview recent advances in AFM methods to directly observe single native GPCRs in membranes and to quantitate their interactions with the environment in physiologically relevant conditions.

## Single-molecule imaging of GPCRs in native membranes

Providing resolution and signal-to-noise ratio far superior to optical microscopes, AFM allows single membrane proteins to be directly observed at subnanometer resolution without labeling or staining. Rhodopsin in the native membrane of discs of rod outer segments (ROS) was the first GPCR imaged by high-resolution contact mode AFM in physiological buffer (Figure 1b, c). Unexpectedly, rhodopsins were observed to dimerize and to arrange in paracrystalline arrays [18^••^]. Further AFM imaging showed that rhodopsins in human, bovine and murine ROS assemble as dimers arranged in rows forming nanodomains [19,20]. This assembly, also confirmed by cryo-electron tomography [21], was suggested to take functional roles in facilitating signal transduction.

## Multiparametric imaging of GPCRs

**Figure 2:**
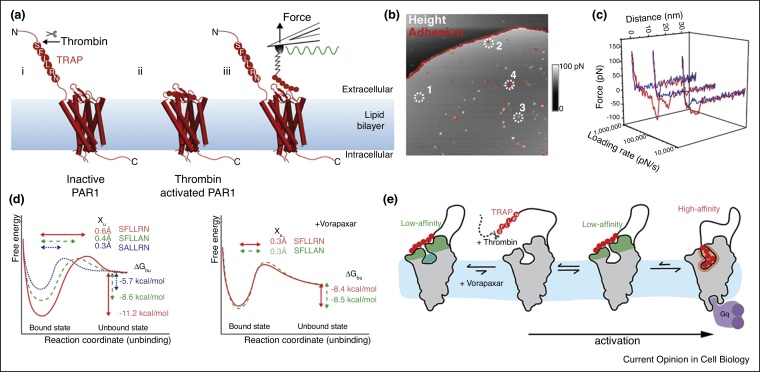
Ligand-binding energy landscape of PAR1 by FD-AFM imaging. **(a)** (i) Cleaving the N-terminal domain of PAR1 by thrombin exposes the SFLLRN sequence, which acts as a tethered ligand of PAR1 (ii). (iii) An AFM cantilever tip functionalized with the short peptide SFLLRN to detect interaction forces with PAR1 by FD-AFM. Sinusoidal oscillation motion of the cantilever is shown by the wavy green line. **(b)** Collecting FD curves pixel-by-pixel in a defined grid pattern provides both, the height and the adhesion information, shown here overlaid on each other. **(c)** Representative FD curves recorded at different loading rates. The force peak denotes specific interactions between the peptide and PAR1. **(d)** Free-energy landscape showing the changes in *x*_u_, Δ*G*_bu_, because of TRAP binding: left panel without and right panel with the antagonist vorapaxar. **(e)** Model depicting the binding of the native SFLLRN ligand (red) to PAR1. For both the vorapaxar-bound and unbound states of PAR1, SFLLRN first binds with low affinity to the extracellular PAR1 surface. Vorapaxar in the binding pocket probably imposes steric hindarance to the peptide binding. However, in the absence of vorapaxar, SFLLRN can access the binding site with high-affinity, activating PAR1 and facilitating the binding of G-proteins [29^••^].

**Figure 3:**
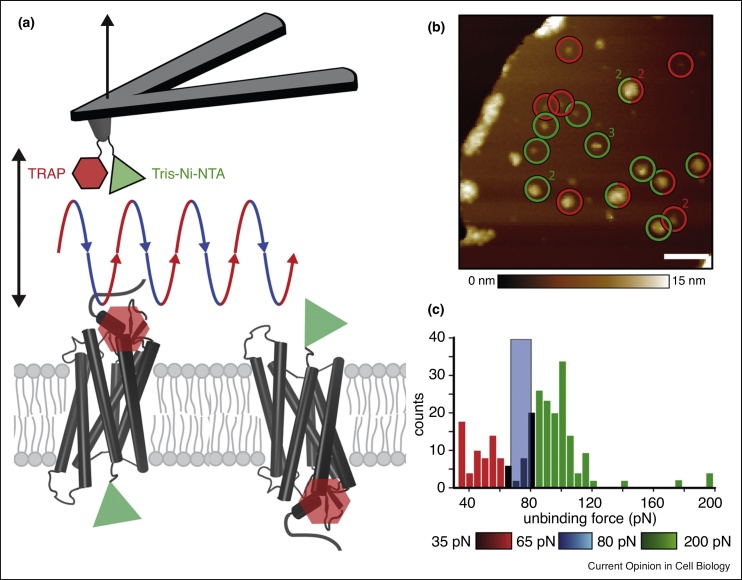
FD-AFM imaging of PAR1 with two ligand binding. **(a)** The AFM cantilever tip is functionalized with two different ligands to simultaneously localize and map binding interactions on PAR1 in the membrane. The cantilever is moved in a sinusoidal motion approaching (blue descending part) and retracting (red ascending part) the ligands from PAR1 molecules. PAR1 can insert in the membrane either for TRAP to bind in the extracellar pocket (red hexagon) or tris-Ni-NTA to bind to His_10_-tag (green triangle). **(b)** PAR1 in a proteoliposome membrane showing sites of specific interactions between SFLLRN (TRAP)–PAR1 (red circles) on the extracellular surface and tris-NTA–His_10_-tag (green circles) on the intracellular surface. The numbers (2, 3) indicate that the specific interactions were detected two or three times during imaging. Scale bar, 80 nm. **(c)** The binding strength of both the ligands can be differentiated (demarcated by the blue bar) as shown by the distribution of rupture forces of SFLLRN–PAR1 (red) and of tris-NTA–His_10_-tag (green) bonds [30^•^].

Conventional AFM imaging modes — contact (a scanning cantilever tip is in constant contact with the protein surface) and non-contact (the tip touches the protein intermittently or not at all) modes — provide information limited to the surface topography of a protein. The recently introduced force-distance (FD) curve-based AFM (FD-AFM) can directly image single TMPs at subnanometer resolution and simultaneously measure multiparametric physical and chemical properties [22,23]. FD-AFM imaging simply records FD curves as the AFM cantilever tip approaches and withdraws from the sample surface while scanning along a high-resolution raster (Figures 2a and 3 a). Each of the thousands of FD curves quantifies the interaction forces between the tip and the sample on a high-resolution topograph. The sensitivity of the approach is sufficient to contour single membrane proteins and their substructures including polypeptide loops [24,25]. Operated in time-lapse mode, FD-AFM can observe single TMPs at work [26,27] or their diffusion and supramolecular assembly [22, 23, 24] at subnanometer resolution [28]. Importantly, to address the complexity of TMP function, FD-AFM allows the topography to be recorded and mechanical, chemical, and biological properties to be mapped simultaneously.

## Imaging GPCRs and quantifying their ligand-binding free-energy landscape

Conventionally, SMFS charts the ligand-binding free-energy landscape by mechanically separating single ligands bound to receptors over several decades of loading rates [26]. In FD-AFM, the ligand is tethered via a flexible PEG-linker to the AFM tip which allows both, contouring the membrane receptor with the tip and separating the ligand from the receptor while mechanically stretching the linker by withdrawing the cantilever (Figure 2a). By applying a sinusoidal motion of the AFM tip, the ligand–receptor bond is ruptured at a wide range of loading rates providing sufficient data to reconstruct the free-energy landscape. This FD-AFM approach was first applied to the human protease-activated receptor 1 (PAR1), a thrombin-activated GPCR, and a key player in coagulation, hemostasis, thrombosis and inflammation [29^••^,30^•^]. Thrombin cleaves the N-terminal exodomain of PAR1 to expose the thrombin receptor-activating peptide (TRAP). TRAP, remaining tethered to PAR1, binds to the extracellular side of PAR1 activating the receptor and triggering downstream signaling cascades (Figure 2a).

To understand the activation mechanism at molecular level, Alsteens *et al.* designed a system to mimic TRAP binding to single PAR1 molecules [29^••^]. Only the SFLLRN sequence of TRAP was tethered to the cantilever tip via a PEG-linker and FD-AFM imaging of PAR1 performed. Approaching the cantilever to the membrane enabled binding of the SFLLRN peptide to PAR1, while retracting the cantilever gave FD curves recording the mechanically induced unbinding process (Figure 2b,c). SFLLRN-PAR1 bonds ruptured between 40–150 pN at loading rates from 4 to 1100 nN/s. The distance, *x*_u_, of the transition state barrier from the PAR1-ligand bound state was 0.6 Å. The equilibrium binding free-energy, Δ*G*_bu_, was −11.22 kcal/mol corresponding to a dissociation constant *K*_d_ of ∼350 nM (Figure 2d), agreeing with the EC_50_ (half-maximal effective concentration) of ∼800 nM from platelet aggregation assays. Changing the native SFLLRN ligand to SFLLAN changed Δ*G*_bu_ to −8.61 kcal/mol and increased *K*_d_ to 30 μM, demonstrating that FD-AFM can detect binding changes introduced by single amino acids. Another mutated peptide SALLRN changed Δ*G*_bu_ to −5.73 kcal/mol and increased *K*_d_ to 3500 μM. These results demonstrated that arginine and phenylalanine in the SFLLRN sequence are crucial for high-affinity interactions with PAR1 consistent with previous functional studies [31,32].

## Quantifying ligand-binding in the presence of an antagonist

An example of directly characterizing ligand–receptor interactions in the presence of an antagonist (or agonist) is PAR1 complexed with vorapaxar. This antagonist attenuates platelet activation, considerably changed the equilibrium binding free-energy, Δ*G*_bu_, of SFLLRN to the receptor to −8.38 kcal/mol and the affinity of SFLLRN from ∼350 nM to ∼40 μM (Figure 2d) [29^••^]. However, the binding-strength and binding-affinity of the SFLLAN peptide to PAR1 remained similar in the presence of vorapaxar suggesting that SFLLAN does not interact with the ligand-binding pocket occupied by voraxapar [7^••^]. A possible explanation could be that vorapaxar sterically hinders the high-affinity SFLLRN-binding to PAR1 as indicated by the shorter *x*_u_ and higher Δ*G*_bu_ but does not block the low-affinity SFLLAN-binding site (Figure 2e).

## Differentiating binding of two ligands

A milestone in the development of functional FD-AFM imaging of GPCRs was to simultaneously quantify the binding of two different ligands to PAR1 (Figure 3). Such differentiation would be important to study how ligands compete during binding to the same receptor. Binding of an artificial ligand tris-*N*-nitrilotriacetic acid (tris-NTA) to PAR1 (with a His_10_-tag) and binding of the natural ligand SFLLRN were simultaneously characterized by FD-AFM imaging (Figure 3) [30^•^]. In general, functionalizing the cantilever tip with ligands for FD-AFM of GPCRs serves multiple purposes: first, single membrane receptors can be imaged at a high-resolution; second, ligand-binding free-energy landscapes of the native receptor can be simultaneously assayed; third, competitive binding of multiple ligands can be detected; and fourth, the information can be quantitatively assessed depending on the receptor’s assembly and functional state, presence of chemical compounds, and membrane composition. Recently it was demonstrated that FD-AFM can also be applied to localize membrane receptors and to simultaneously characterize their free-energy landscape in living mammalian cells [33^•^]. We envision that the FD-AFM approach will be ready to study ligand-binding to GPCRs in living cells and to characterize how the binding depends on and alters the cell state.

## Single-molecule force spectroscopy of GPCRs in membranes

AFM-based SMFS characterizes the mechanical and kinetic stability of the structural intermediates of GPCRs at the resolution of single α-helices [34] or a few amino acids [35^•^]. In a nutshell, the tip of the AFM cantilever acts as a ‘sticky finger’ to pick up single membrane receptors from either terminal end. Pulling on the terminus by retracting the cantilever applies a mechanical force on the receptor, stepwise unfolding and extracting the receptor from the membrane [34,36]. This stepwise unfolding describes highly reproducible unfolding structural intermediates of the GPCR. The FD curve recorded is a unique fingerprint of the mechanical and kinetic stability of the GPCR. The force peak pattern of these curves reacts sensitively to alterations in intramolecular and intermolecular interactions stabilizing a GPCR, such as different conformational states or subtle physical and chemical changes including pH, assembly with other proteins, ligand-binding, mutations, and lipid membrane composition.

## Bovine rhodopsin: unfolding a GPCR paradigm

Bovine rhodopsin from ROS discs was the first GPCR characterized by SMFS [37]. It was shown that the conserved disulfide bridge (S–S) between cysteines 185 and 187 stabilized almost all the structural segments of rhodopsin. Furthermore, Zn^2+^ binding at a putative extracellular site increased the mechanical stability of bovine rhodopsin and, supported by *in silico* results, suggested to favor rhodopsin dimerization [38^••^]. These early results led to further investigation of the mechanisms that (de-)stabilize GPCRs in conditions simulating (mal-)functional states.

The crystal structure of opsin shows clear differences in helical arrangements compared to dark-state rhodopsin [5^••^,^39^]. Apoprotein opsin (without 11-*cis*-retinal) shows a low constitutive activity [40] leading to retinal degeneration in Leber congenital amaurosis [41]. To understand how the native inverse agonist, 11-*cis*-retinal, modulates the energy landscape of rhodopsin, single rhodopsins were unfolded from the ROS discs of *Rpe65−/−* mice unable to synthesize 11-*cis*-retinal, and compared to the native dark-state rhodopsin from wild-type mice [42^•^]. The mechanical forces stabilizing the unfolding structural intermediates of opsin were higher than those stabilizing dark-state rhodopsin.

Unfolding a GPCR over a wide range of loading rates provides parameters, including the unfolding rate, *k*_0_ (reciprocal of lifetime), and the distance, *x*_u_, between the native and transition states that define the free-energy landscape of a GPCR conformation [34]. Overall, structural segments of opsin exhibited lower lifetimes (higher *k*_0_) and lower unfolding free-energy, Δ*G*_u_^*^, in the range 20.6–24.5 *k*_B_T compared to 21.5–38.0 *k*_B_T for dark state rhodopsin. Furthermore, the structural segments of opsin were stabilized by narrower free-energy valleys (estimated by *x*_u_) signifying the restriction of conformational states. Similarly, G90D rhodopsin, which also exhibits constitutive activity, exhibited narrower free-energy valleys, lower lifetimes and unfolding free-energy barriers compared to wild-type rhodopsin [43]. Because both opsin and G90D rhodopsin are constitutively active, the insight gained by SMFS may signify a trend of how interactions change in native and constitutively active states. The higher energetic stability of dark state wild-type rhodopsin may lock the receptor in the inactive state which may be required for maintaining low noise and single photon sensitivity in rod photoreceptor cells [44].

## Energy landscape of β_2_-adrenergic receptor bound to agonist and antagonist

Nuanced changes in the free-energy landscape of human β_2_-adrenergic receptors (β_2_AR) upon agonist-binding and antagonist-binding were determined by SMFS. The energy landscape of β_2_AR was characterized in the apo-state and in the presence of the synthetic agonists BI-167107 (BI, Boehringer Ingelheim) and THRX-144877 (THRX, Theravance), the natural agonist adrenalin, the inverse agonist carazolol, and the neutral antagonist alprenolol [45]. The distance from the folded state to the transition state (*x*_u_) of every structural segment of apo-β_2_AR ranged from 0.3 to 0.6 nm. Agonists and carazolol-binding increased the *x*_u_ of the structural core comprising helices III and IV, which hosts the ligand-binding sites, from 0.55 nm (unliganded β_2_AR) to 0.73 nm (THRX), 0.71 nm (BI), 0.65 nm (adrenalin), and 0.79 nm (carazolol). The agonists and the inverse agonist carazolol significantly increased the kinetic stability of the core structural region. The magnitude of stability increases correlated with ligand affinity — lowest values were observed for the highest affinity ligands. In unliganded β_2_AR, Δ*G*_u_^*^ of the structural segments ranged from 20 to 23 *k*_B_T. Ligand-binding to β_2_AR increased the Δ*G*_u_^*^ of the structural core by 7.7 *k*_B_T (BI), 6.9 *k*_B_T (THRX), 3.2 *k*_B_T (adrenalin), and 7.6 *k*_B_T (carazolol). These results suggest that the structural core comprising helices III and IV resides on a rough energy landscape, which is populated by multiple conformations amenable to bind different ligands [46]. Once a ligand binds, the structural core is stabilized in a deep energy well that helps to tune β_2_AR activity.

## Molecular changes in PAR1 with anti-platelet agent vorapaxar

**Figure 4:**
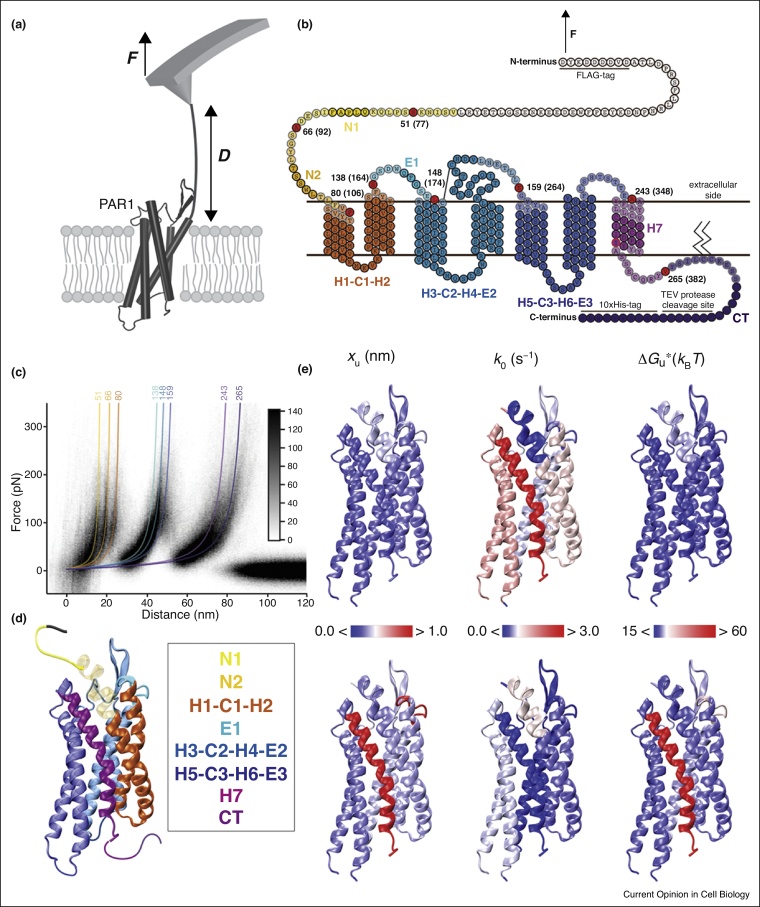
SMFS can differentiate the functional states of single PAR1 molecules. **(a)** The AFM cantilever tip is non-specifically attached to a PAR1 terminal and retracted to apply a force thereby unfolding and extracting the protein from the membrane. This unfolding and extraction process is recorded by a FD curve. **(b)** Under the external force applied by the tip, the receptor unfolds in discrete steps; the structural segments that unfold individually are shown in different colors on the PAR1 secondary structure. **(c)** The structural segments are determined from worm-like chain (WLC) curves (colored lines) fitting the force peaks of FD curves. Above the WLC curves, the contour lengths of the unfolded polypeptide are denoted in amino acids. The reproducibility of the unfolding process is determined by superimposing the FD curves. **(d)** Structural segments mapped onto the tertiary structure of PAR1 in the presence or absence of a ligand reveal the underlying molecular changes. **(e)** Heat maps of the energy landscape parameters of unliganded and vorapaxar-bound PAR1 mapped on the tertiary structure of PAR1 (PDB: 3VW7) [12^••^]. Figures have been adapted from Ref. [47].

More recently, PAR1 was unfolded in the presence of vorapaxar to further understand the common trends in GPCR activation and inhibition [47]. PAR1 comprises structural segments (or intermediates) similar to those observed in rhodopsin and β_2_AR (Figure 4). Binding of vorapaxar increased the conformational variability (estimated by *x*_u_), lifetime (reciprocal of *k*_0_), unfolding free-energy, ΔG_u_^*^, and mechanical flexibility of most structural segments (Figure 4e). Furthermore, while PAR1 in the unliganded state was found to reside in a rough free-energy valley populated by many small energy wells, vorapaxar-binding smoothened the energy valley [47]. Smooth energy valleys are thought to reduce the structural variability [48], which combined with kinetic stabilization restrict PAR1 to inactive conformations, all highlighting that vorapaxar is an effective antagonist. The changes observed in PAR1 upon antagonist-binding are consistent with the trends observed on mouse opsin, dark-state rhodopsin [42^•^] and on human β_2_AR in the presence and absence of various ligands [45]. These insights may thus highlight common mechanisms of how class A GPCRs are structurally stabilized in various states.

## Role of cholesterol on GPCR stability

Single β_2_AR proteins mechanically unfolded in the presence and absence of a cholesterol analog, cholesteryl hemisuccinate (CHS), showed similar structural intermediates [49^•^]. However, CHS increased the mechanical and kinetic stability of most β_2_AR structural segments except the core region which hosts the ligand-binding sites. In the presence of CHS, the structural intermediates were stabilized marginally and resided in energy wells 1.5–3.9 *k*_B_T deeper with a consequent 4–50 fold increase in kinetic stability. A more stable β_2_AR in a membrane with CHS suggests a role for cholesterol and lipid composition in modulating GPCR function [49^•^].

## Outlook and vision

The possibility to observe individual GPCRs and to sense their interaction with ligands presents new opportunities in assessing interactions of GPCRs with G-proteins and arrestin in signal transduction. The capability of the AFM to localize ligand-binding events on single receptors, combined with an exceptional signal-to-noise ratio at subnanometer resolution, will allow to study changes in interactions between receptors and ligands, other molecules or proteins in dependence of their macromolecular environment. New developments to map ligand-binding events in living mammalian cells while simultaneously observing the cells by advanced optical microscopy techniques may soon address how GPCR states are modulated in dependence to the cell state and *vice versa*.

Mapping intermediates on the complex energy landscape of different GPCR functional states opens avenues to investigate dynamic modulation of GPCR with ligands and inhibitors. Correlating the information with functional assays may provide a more reliable basis of controlling GPCR activity with pharmacological chaperones in health and disease. This will be a focus in the future to determine the stability of GPCRs in complex with G-proteins and arrestin [50].

## Conflict of interest statement

Nothing declared.

## References and recommended reading

Papers of particular interest, published within the period of review, have been highlighted as:

• of special interest
•• of outstanding interest
